# A Theory as to the Etiology of Pyorrhea Alveolaris*Reprinted from *The Dental Record*, October 1, 1920.

**Published:** 1920-11

**Authors:** Stanley Colyer

**Affiliations:** M.D.Lond. Elliot, S. Africa


### A THEORY AS TO THE ETIOLOGY OF PYORRHEA ALVEOLARIS *

BY STANLEY COLYER, M.D.LOND., ELLIOT, S. AFRICA

In the course of my investigations into the dental conditions among certain African tribes, investigations pursued chiefly with the object of discovering the real causes of dental caries, a curious distribution of the disease known, most generally, as pyorrhea alveolaris came to my notice. I observed that among the Barotzi along the Upper Zambesi, and other tribes living in the neighborhood of Lakes Mweru and Bangweulu, that whilst pyorrhea alveolaris was a common disease, dental caries was comparatively uncommon, whereas among natives living in the Transkeian territories (S.E. Africa), the reverse was the case. On the assumption that dental caries was a food disease, the deduction, after a consideration of the various dietaries, was that it was due chiefly to the inclusion of commercial sugar into the diet of the latter.

Now it is possible, assuming that pyorrhea alveolaris is also a food disease, to show that there is a certain fundamental difference in the food of these natives which may be, and probably is, the cause of this condition. Having found this essential factor, in order that it may be exalted to the position of one of world-wide importance, it must be shown that the pathological state that it brings about may be likewise induced by the food of all people suffering from the disease.

The pyorrhea alveolaris of which I speak is the general chronic periodontitis with accompanying changes in the structure of the alveolus and of the teeth. It is progressive and possibly differs in its origin from other cases that may be due to direct infection, following injury, or

* Reprinted from *The Dental Record,* October 1, 1920.

in general septicemic states. It begins, so dental surgeons say, as a marginal gingivitis, and its earliest clinical evidence is a destruction of the interdental papilla. *It does not, however, follow that the interdental papilla is the first part of the gingival margin to be attacked.* It is a widely spread disease and though it may occur independently of civilization it follows closely its movements. Wild animals when brought under domestication suffer, though in a state of nature they are but rarely attacked.

It is fair, then, to assume that there is some underlying condition brought about by the modern ways of living which is common to civilized and uncivilized beings as well as to domesticated animals which will account for this disease. Elsewhere I have shown from certain theoretical considerations that dental caries, most probably, arose after the discovery of cooking, and I endeavor to prove in this paper that pyorrhea alveolaris also dates its origin from the same epoch, and that it is dependent upon the quality of the food consumed. To do so I propose to commence by reconsidering the foodstuffs of the tribes I have studied in Africa.

LIST OF FOODSTUFFS

| Barotzi, and Natives near Lakes Mweru and Bangweulu. | S. E. African Natives (E. Griqualand). |
|---|---|
| I. Grain. | |
| (*a*) Indian corn (mealies) | (*a*) Indian corn (mealies) |
| (*b*) Millet, of various kinds | (*b*) Millet—in small quantities |
| (*c*) Kaffir corn, varieties of | (*c*) Kaffir corn, varieties of |
| (*d*) Wheat, very small quantities | (*d*) Wheat in small quanties |
| (*e*) Rice, very small quantities | (*e*) Rice, imported only |
| II. Vegetables. | |
| (1) *Above ground* | (1) *Above ground* |
| (*a*) ———— | (*a*) European potatoes |
| (*b*) Beans and peas of various kinds (seeds only eaten) | (*b*) Beans and peas of various kinds (seeds only eaten) |
| (*c*) Pumpkins | (*c*) Pumpkins |
| (2) *Underground* | (2) *Underground* |
| (*a*) Cassava | (*a*) ———— |
| (*b*) Monkey nuts, in fairly large quantities | (*b*) Monkey nuts, in small quantities |
| (*c*) Sweet potatoes and allied corns, large quantities | (*c*) Sweet potatoes, etc., very scarce |

| | |
|---|---|
| III. FLESH FOODS. | |
| (*a*) Oxen, sheep, goats, rats, etc., and that of game, animals and birds | (*a*) Oxen, sheep, goats and chickens (game, killed out) |
| (*b*) Caterpillars, insects, flying ants, etc. | (*b*) Caterpillars, etc., said to be eaten raw by Kaffirs only |
| (*c*) Fish—mostly dried, in fair amount | (*c*) *Fish*—very scarce |
| IV. SAVOURIES AND RELISH FOODS. | |
| (*a*) Leaves of wild spinach, pumpkin, cassava, etc. | (*a*) Leaves of pumpkin, potato, and other plants |
| (*b*) "Salt" in small quantities in some districts, large quantities in other | (*b*) "Salt," all purchased from stores |
| (*c*) Sugar cane and honey in small amounts | (*c*) Sugar cane very rare |
| (*d*) Sugar, consumed by rich only and not much used | (*d*) *Sugar*—a common article of diet |
| (e) —————— | (*e*) Pepper and curry powder |
| (*f*) Roots of certain kinds in season | (*f*) Roots, etc., (eaten raw by Kaffirs) |
| V. DRINKS. | |
| (*a*) Kaffir beer, either fermented or unfermented | (*a*) Kaffir beer, either fermented or unfermented |
| (*b*) Milk—fresh and curdled | (*b*) Milk—fresh and curdled |
| (*c*) Water | (*c*) Water |
| (*d*) Tea and coffee—rare | (*d*) Tea and coffee in large quantities |

I have already said that pyorrhea alveolaris among the Transkeian natives (S.E. Africa) is comparatively uncommon, whereas among the Barotzi and those dwelling near Lakes Mweru and Bangweulu it is rampant. The extent of the disease among the Mweru and Bangweulu natives will be evident from the figures that I collected from one thousand male natives examined successively. Three decades of life are dealt with and the extent of the disease is divided into three degrees: (1) all cases showing early suppuration—nearly always around the anterior teeth—to complete but not severe infection of the whole set; (2) severe and incurable cases; (3) advanced cases with marked loosening and loss of teeth.

| Age | Number | Free of the Disease | 1st degree | 2nd degree | 3rd degree |
|---|---|---|---|---|---|
| 10—20 years ......... | 246 | 8 | 225 | 12 | 1 |
| 20—30 years ......... | 670 | 35 | 586 | 47 | 2 |
| 30—45 years ......... | 84 | 0 | 51 | 25 | 8 |
| | 1,000 | 43 | 862 | 84 | 11 |

(I regret that I have no figures for the Barotzi or the Transkeian natives.)

A glance at the list of foodstuffs would appear to show no great difference between those of the respective people under consideration. The S.E. African natives are not so careful in the winnowing of their grain, and are fond of eating cooked mealies which are merely bruised, but I do not think that these differences are sufficient to account for their comparatively remarkable freedom from pyorrhea alveolaris. I think, rather, that the essential difference lies in the fact that they do not possess the root known as Cassava. This root, when dried, crushed and prepared for food, forms an unpleasant smelling, stodgy, elastic and rather sticky mass. It is the main food of the Barotzi, especially of those living away from the Zambesi, and it is considered by the Barotzi themselves to be the cause of the present state of their teeth—a view with which I certainly agree. This root, cassava, is grown and freely eaten by the people around Lakes Mweru and Bangweulu, and their teeth are comparable in all ways to those of the Barotzi. If we go further east we come to the parts where it ceases to be grown and there is a corresponding improvement in the teeth.

Both the Barotzi and the S.E. African tribes are rich in cattle. Among the latter it is more evenly distributed so that proportionately more people eat meat than among the former, and as there is no question that they do not suffer from the disease as severely as the Barotzi, it is difficult to attribute the cause of the disease to meat-eating.

Moreover, it must never be forgotten that putrefactive and proteolytic changes take place in epithelial tissue shed from the mucous membrane of the mouth and that this process appears to be the normal method of removing waste tissue in interdental spaces.

In the last edition of *Dental Surgery and Pathology*, by my brother, it is there stated (page 565) that "the evidence points to the disease being started by injury of the gingival margin from food debris, or by the local action of toxins as seen in the marginal gingivitis of mouth-breathers.

"The prevalence of the disease is probably due to the character of the diet of the present day. Much of our food is now prepared in such a manner that it readily accumulates around the teeth and is of a character which easily undergoes fermentation." Later in the same work it is explained that the difference between caries and periodontal disease is that caries is due to enzyme action on carbohydrates, periodontal disease to enzyme action on proteins. "Made-up dishes" are supposed to be worse than "plain, fresh-cooked food," but for what reason is not stated. Lastly, imported frozen meat is added to the list of "suspect" foodstuffs because it undergoes putrefactive changes more easily than fresh animal food. It is thus clearly laid down that the disease is due to the local action of toxins formed from animal protein which has either been introduced into the mouth or consists of epithelial debris—waste cells of the mucous membrane. The important question as to why modern meat should cause this disease is answered by saying that meat is frequently consumed as "made-up" dishes and imported frozen meat is more liable to undergo putrefactive changes than freshly-killed meat. *Putrefactive changes in protein debris is obviously considered to be at the base of this condition.* Without adducing arguments I will content myself with saying that I consider this view to be wrong, and I believe that though protein may be in some slight degree responsible for bringing about the earliest stages of this disease, the chief role must be assigned to the carbohydrates, and that bacteria play no part until the disease is established—clinical pyorrhea being of the nature of a secondary infection.

I showed many years ago (1904) that soft food *per se* was not the cause of dental caries, if for no other reason than that it could not explain the position at which the earliest stages of interstitial caries occurred. Likewise the decomposition of soft food debris at the cervical edge can not satisfactorily explain the distribution of marginal gingivitis which is *general to a few teeth and not local except in cases of definite injury.* In the case of dental caries my deduction from the evidence was that the food that underwent acid fermentation was *in a state of solution* and was held interstitially by capillary attraction; so in the case of marginal gingivitis the evidence leads me to the conclusion that the disease is due to food in solution, held at the festoons of the gum margin and in the interstitial spaces by the same force. It is not necessary for me to prove that the space between the gum margins and the teeth, and between adjacent teeth are capillary spaces, but any one who has a doubt should wash his mouth out with a colored and rather viscous fluid, and subsequently examine his teeth in a mirror. He will then see the margins of his gums clearly outlined by the fluid.

I have stated in an earlier part of this paper that the essential difference between tribes markedly affected by pyorrhea and those who are not, is the presence in the food of the former of a sticky, glutinous food known as cassava. It is this quality of stickiness in the food whereby it is enabled to be held in capillary spaces, and owing to its indiffusibility with the saliva, to remain in position for some time, that would seem to determine the disease. Mere softness of food, neglect of oral hygiene and the consumption of animal food, none of these seem to have a clear claim.

It is my contention, then, that it is chiefly the cooked carbohydrate food which is held by capillary attraction in the festoons of the gums and the interdental spaces which lead to the earliest stages of marginal gingivitis which, I will endeavor to show, is primarily due to physical damage to the superficial cells and not to bacterial destruction of waste food material. The theoretical proof of my contention is by no means an easy matter and involves an incursion into modern physiology which I am fearful of taking; but if any theory is to have any support other than what I have drawn from clinical observation, I am compelled to make it.

Evolutionary history would seem to show that an animal which has evolved to its environment remains in health so long as its environment does not change. Any change that may occur calls upon the adaptability of the animal, and any failure to adapt itself spells disease and possibly extinction. In diseases that have led to the destruction of animals in the past, dental disease may have played its part, for it is said that certain fossil remains of minosaurs and plesiosaurs of the cretaceous era were affected with general periodontitis. So man, we may assume, evolved more or less perfectly adapted, but being of a wandering and insurgent nature, was constantly changing his environment. Disease was the natural outcome, and of these there are few more common than the one under consideration. Cooking was one of the most important environmental changes to which man had to adapt himself, and I wish to draw attention to certain changes that it brings about in food which, in my opinion, are the cause of general periodontal trouble.

The effect on carbohydrates is to break up the starch grains by bursting the cellulose capsule, thus freeing the granulose (starch particles) which are partly soluble in hot water—producing when concentrated what is known as mucilage of starch.

The effect on animal food is to convert the insoluble collagen of connective tissue into *soluble gelatine.*

From my point of view the important change that cooking has brought about is that it produces a more gelatinous kind of nutriment. It is obvious that such food is more viscous and adhesive and, if once established in capillary spaces, will not readily be removed by diffusion with saliva. It thus remains in contact with the marginal epithelium and is not washed away as is uncooked food. It is this prolonged contact which leads to damage to the superficial cells which is the first stage of marginal gingivitis, the damage being of a physical and not a bacterial nature.

Now, ordinarily speaking, a cell remains normal if it is surrounded with an isotonic solution which does not damage the cell membrane. If the solution is not of this nature a damage to the membrane occurs which increases its permeability. And here it may be noted as a matter of some dental interest that of all non-electrolytes used in physiological experiments cane sugar in isotonic solution appears to be the least injurious to the cell membrane, though in time it will increase its permeability. Consider now the influence of saliva on the mucous membrane. As saliva is of variable constitution it can not always be isotonic with the cell contents of the mucous membrane, but, as these remain normal under its influence, there must be something in the saliva which keeps intact the cell membrane and so prevents undue osmosis. This substance is *calcium*, and there is strong physiological evidence to show that calcium is a cell restorative and capable of rendering a cell membrane made permeable once again impermeable. It thus follows that when saliva can circulate freely over the mucous membrane of the mouth it can restore that tissue should its cell-impermeability have been temporarily upset by contact with substances rendering it more permeable. In pre-cooking days, when the majority of the food taken was of a non-viscous nature, its contact with the mucous membrane was momentary, and any damage it may have inflicted was immediately remedied by the salivary wash.

It follows from the above that food may act in three distinct ways upon the mucous membrane:

(1) By contact it may damage the cell membrane, leading to an increased permeability of the cell wall;

(2) Prolonged contact allows of abnormal osmotic interchanges either towards or away from the cell, depending upon whether the food solution is hypotonic or hypertonic towards the cell contents.

(3) It prevents the wash of the saliva and so interferes with the restorative action of the calcium.

A pathological condition is thus established which, if continued, must lead to the destruction of the superficial cells, which in their turn will affect subjacent cells. Their dying and dead bodies become irritant by virtue of chemical products of the disintegration of the protoplasm brought about by autolysis, and thus an inflammation of a non-bacterial type is induced. Bacterial infection is grafted upon the above state, and a disease which is induced by physical cause is continued as a bacterial infection.

The prevention of the disease would seem to be simplicity itself, for the essential thing is merely to get rid at the end of each meal of that dangerous layer of food material held at the gum margins. To reach that end we may return to pre-cooking days and eat only of the natural products of Mother Earth, or, if we would rather adapt ourselves to the conditions that have arisen, we may follow the custom of certain natives who massage their gums and swill out their mouths after each meal, or we may adopt the probably less efficient methods of eating acid fruit or take salivary stimulants, or we may carefully cleanse our teeth and gums by the judicious sucking of our teeth—a process one might call auto-cleansing.

There, for the present, I leave this theory to the consideration of those whom it may interest, in the hope that it may open up a new line of approach along which this important disease may be attacked.

NOTES.—Full particulars of the foodstuffs of the African tribes under consideration will be found in the papers referred to below.

CASSAVA (better known as manioc). The Indian varieties contain about 1 per cent. of proteids and of fats, 15-30 per cent. of starch. Tapioca is a refined product of cassava and contains about 86 per cent. of starch.

REFERENCES

(1) *Principles of General Physiology.* W. M. Bayliss, 1918.
(2) *Dental Caries among certain Central African Races.* S. Colyer. *Dental Record,* Vol. XXXVI, page 1.
(3) *Dental Disease among Certain Central African Tribes.* S. Colyer. *Dental Record,* Vol. XXXVII, page 405.
(4) *Dental Caries among Certain S.E. African Tribes.* S. Colyer. *Dental Record,* Vol. XXXIX, page 401.
(5) *Dental Surgery and Pathology.* Sir J. F. Colyer.

---

TO PREVENT FAINTING UNDER NOVOCAIN ANESTHESIA. For the benefit of those who are interested in novocain and for those who are about to take up its use, I may say that for a number of years it has been my practice to give my patients a half teaspoonful of aromatic spirits of ammonia in half a glass of water ten to fifteen minutes before injecting the novocain solution. In following this plan I have not been delayed, have had practically no symptoms of fainting, and the results altogether have been most satisfactory to myself and my patients.

Aromatic spirits of ammonia is a perfectly harmless preventive and I can see no reason for waiting until the patient shows signs of fainting before administering the drug.

I am sure if the members of the dental profession who are using novocain will try the plan I have suggested, they will be more than pleased with the result.—H. M. Beck, in *Cosmos.*

